# Structural and electrophysiological dysfunctions due to increased endoplasmic reticulum stress in a long-term pacing model using human induced pluripotent stem cell-derived ventricular cardiomyocytes

**DOI:** 10.1186/s13287-017-0566-6

**Published:** 2017-05-11

**Authors:** Chang Cui, Le Geng, Jiaojiao Shi, Yue Zhu, Gang Yang, Zidun Wang, Jiaxian Wang, Minglong Chen

**Affiliations:** Division of Cardiology, The First Affiliated Hospital of Nanjing Medical University, Nanjing, 210029 China

**Keywords:** Pace, Induced pluripotent stem cells, Electrophysiology, ER stress

## Abstract

**Background:**

Long-term ventricular pacing has deleterious effects and becomes more significant when cumulative percent ventricular pacing (Cum%VP) exceeds 40% of time. However, cellular disturbances and pathways by which pacing leads to myocardial disorders are not well understood. Attempts to resolve these questions have been hampered by difficulties in obtaining human cardiac tissue and the inability to build a longer-lasting (lasting longer than weeks) pacing model in vitro.

**Methods:**

Human induced pluripotent stem cell-derived ventricular cardiomyocytes (VCMs) were cultured in the presence of electrical stimulation for 2 weeks. Quantitative structural and electrophysiological analyses were used to define the functional disturbances of pacing.

**Results:**

Compared to controls, paced VCMs exhibited a remarkable reduction in the contractile protein expression, an increased apoptosis ratio and electrophysiological remodelling in a Cum%VP-dependent manner. Investigation of the protein expression levels revealed that long-term pacing universally activated both ER stress and downstream calpain. Moreover, the inhibition of calpain attenuated the adverse effects on the structural remodelling and increased the I_Ca, L_ in paced VCMs.

**Conclusions:**

The results demonstrated that pacing VCMs for 2 weeks in vitro led to a series of structural and electrophysiological dysfunctions. The increased ER stress and downstream calpain could be a central mechanism underlying the disease pathogenesis. This finding could represent a new therapeutic target in the management of long-term pacing patients.

**Electronic supplementary material:**

The online version of this article (doi:10.1186/s13287-017-0566-6) contains supplementary material, which is available to authorized users.

## Background

Cardiac pacing has been an effective treatment in the management of patients with bradyarrhythmias and tachyarrhythmias [1]. In cardiac pacing, the endocardial pacing lead is typically positioned at the right ventricular (RV) apex. However, several implantable cardioverter-defibrillator (ICD) and pacemaker trials have suggested that RV pacing may have detrimental effects on the cardiac structure and left ventricular (LV) function [2, 3] These adverse effects increase the risks of atrial fibrillation (AF) [4], heart failure [5] and death during ICD therapy [6]. Histopathological studies have indicated that long-term pacing could induce significant alterations in the myofibrillar size, fibrosis, fat deposition, sclerosis, and mitochondrial morphological changes [7]. In addition, alterations in potassium and calcium channels likely occurred during this process [8]. While the pathological evidence has identified the pacing-induced cardiac injury, the cellular disturbances and pathways by which pacing leads to myocardial disorders and ventricular arrhythmias are not well understood [9]. Attempts to resolve these questions have been hampered by difficulties in obtaining human cardiac tissue and the inability to build a longer-lasting (lasting longer than weeks) pacing model in vitro.

Here, human induced pluripotent stem cell (iPSC)-derived ventricular cardiomyocytes (VCMs) were cultured in the presence of electrical stimulation for 2 weeks. We studied the morphology of the long-term paced VCMs, quantified the apoptosis ratio using flow cytometry and recorded their electrophysiological properties with patch clamp. Compared to the controls, paced VCMs exhibited a remarkable reduction in the contractile protein expression, an increased apoptosis ratio and electrophysiological remodelling. Western blotting studies revealed that the proteins involved in endoplasmic reticulum (ER) stress were universally upregulated, suggesting a possible regulatory mechanism. Importantly, we demonstrated that treatment with a calpain inhibitor (calpeptin) could attenuate the adverse effects on the structural remodelling and increase the L-type calcium current (I_Ca, L_) in paced VCMs. In summary, these findings demonstrated that long-term pacing could induce adverse effects on the structural and electrophysiological properties of human iPSC-derived VCMs. These adverse effects could be regulated by ER stress and downstream calpain activity, which might represent a new therapeutic target in the management of long-term pacing patients.

## Methods

### Human iPSCs culture and VCMs differentiation

The human iPSC line (NC5) was seeded on six-well plates after Matrigel (Corning, Corning, NY, USA, 354277) coating for 1 hour. Cells were maintained with daily refreshment of the mTeSR™ medium (Stemcell Technologies, Vancouver, Canada, 05850). The ventricular cardiomyocytes were derived from iPSCs as previously described [10–12]. Briefly, hPSCs at 90–100% confluence were cultured in an RPMI 1640 medium (Gibco, Carlsbad, CA, USA, 1744361) containing B-27® supplement minus insulin (1:50, Gibco, A1895601) from day 0–8. Additional CHIR-99021 (6 μM, Selleckchem, Houston, TX, USA, S2924) and IWR-1 (5 μM, Sigma-Aldrich, St. Louis, MO, USA, 10161) were employed on day 0–1 and day 3–4, respectively. BMS493 (1 μM, Sigma-Aldrich, B6688) was added to the differentiating cultures on day 5–7 of differentiation. RPMI 1640 containing B27® supplement (1:50, Gibco, 17504-044) was used from day 8 onwards, and the medium was changed every other day. Cells were incubated in a 5% CO_2_ and 37 °C environment for 6 weeks and paced for another 2 weeks until dissociation for the functional and electrophysiological analyses. Pacing was performed using a C-Pace100™ culture pacer and CDish100™ culture dishes (IonOptix Corporation, Wageningen, Netherlands) with 0.5 ms pulse width, 3 V voltage and 1.2 Hz frequency pulses. During the pacing, cells were treated with calpeptin (5 μM) to inhibit calpain activity.

### Impedance beating recordings

The cardiomyocytes were seeded on Nanion CardioExcyte 96 Sensor Plates (NSP-96, Nanion Technologies, Munich, Germany) after 0.1% gelatin coating for 1 hour. The initial volume per well was 200 μl and contained approximately 50,000 cells. After seeding, the NSP-96 and CardioExcyte 96 were returned to the incubator. The medium was changed every day until the cells formed a synchronously beating network. Then, 100 μl of the medium was changed every day to maintain a regular beating pattern. In this experiment, the cellular beat rate, base impedance and beat amplitude were monitored.

### Cell viability

Cell viability was detected by the Cell Counting Kit (CCK-8) (Dojindo, Kumamoto, Japan, CK04-500) as previously described [13]. In brief, cells were seeded into six-well plates at 2 × 10^5^ cells per well, which differed from previous studies in which cells were seeded into 96-well plates. This distinction was due to the pacing device, which is designed only for six-well plates. Then, VCMs were exposed to 0.5 ms pulse width and 1.2 Hz frequency pulses with 0, 1.5, 3, 4.5, 6 V voltage for 2 weeks. A total of 150 μl CCK-8 was added to each well and incubated at 37 °C for 2 h and then the absorbance at 450 nm was measured with a microplate reader (BioTek, Winooski, VT, USA).

### Transmission electron microscopy (TEM)

The cardiomyocytes were dissociated with trypsin into single cells for the TEM evaluation [14]. Cells were fixed with 2.5% glutaraldehyde for 1 hour and rinsed with phosphate-buffered saline (PBS). After the dehydration and embedment, thin sections were cut and stained with uranyl acetate and lead citrate. Images were obtained under a transmission electron microscope (JEM-1010, Jeol Ltd., Tokyo, Japan) operated at 75 kV. Ultrastructural observations were performed in multiple sites of clearly identified junctional complexes. Mitochondrial swelling has been previously described [15], and we calculated the mitochondria swelling percentage in three biological replicates.

### Immunofluorescent staining

The cardiomyocytes were fixed in 4% paraformaldehyde for 20 min at room temperature, rinsed with (PBS) three times and then blocked with 5% BSA in PBS for 30 min before being subjected to immunostaining with the appropriate primary antibodies for 16 h. The following primary antibodies were included in the immunofluorescent staining: rabbit anti-Cx43 (1:200, Abcam, Cambridge, MA, USA, ab11370) and mouse Anti-Cardiac Troponin T (1:200, Abcam, ab8295). After incubation with the primary antibody, cells were washed with PBS and then incubated for 1 h with the fluorescent-conjugated secondary antibody. The secondary antibodies were goat anti-rabbit IgG (Alexa Fluor®488, 1:500, Abcam, ab150077) and donkey anti-mouse IgG (Alexa Fluor®555, 1:500, Abcam, ab150106). The nuclei were stained with DAPI (ProLong® Gold Antifade Reagent with DAPI, Life Technologies, Carlsbad, CA, USA, P36931). The mounted sections were visualized using a confocal high content screening system (Opera®, PerkinElmer, Waltham, MA, USA), and the images were analysed using ImageJ (National Institutes of Health, version 1.8.0_77).

### Hoechst 33342 staining

The typical morphological features of apoptotic cells were evaluated using Hoechst 33342 staining (Beyotime Institute of Biotechnology, Shanghai, China). After pacing, the cells were washed twice with cold PBS and fixed in freshly prepared 4% paraformaldehyde for 30 min. Cells were then washed with cold PBS again before being incubated with 5 μg/ml Hoechst 33342 for 15 min at 37 °C in the dark. Finally, cells were washed with PBS, and apoptotic cells were identified under a fluorescence microscope (Axio Vert.A1, Carl Zeiss, Jena, Germany). Normal cells showed homogeneous blue chromatin with an organized structure. By contrast, apoptotic cells presented bright blue chromatin, which was highly condensed or fragmented.

### Flow cytometry analysis

Cells were dissociated with trypsin and fixed with 4% paraformaldehyde for 20 min at room temperature. For the intracellular epitopes, cells were permeabilized in PBS containing 0.2% Triton X-100 for 20 min at room temperature. Cells were incubated with rabbit anti-cardiac troponin I (cTnI) IgG (Alexa Fluor® 488, 1:200, ab196384) or mouse anti-MLC2v IgG (PE, 1:20, 130-106-133) in PBS on ice for 30 min. A rabbit IgG antibody (Alexa Fluor® 488, 1:200, IC1051G) or mouse IgG antibody (PE-Texas Red®, 1:200, ab131392) was used as an isotype control. The stained cells were counted using a flow cytometer (BD FACSCalibur BD, Franklin Lakes, NJ, USA). The percentage of positive cells was normalized to the isotype control population in each experiment to accurately determine whether a change in cell population occurred.

Cell apoptosis was also assessed by flow cytometry. After pacing, approximately 1 × 10^6^ cells were harvested, washed twice with pre-chilled PBS, resuspended in 300 ml binding buffer and then incubated with annexin V/propidium iodide (PI) (BD Pharmingen, San Diego, CA, USA) for 20 min in the dark. The samples were analysed using a flow cytometer (BD FACSCalibur) within 1 h.

### Optical mapping

Optical mapping was performed as previously described [16]. Cardiomyocytes were loaded with 4 μM Di-4-ANEPPS (Life Technologies, D1199) for 20 min at room temperature (approximately 20 °C) in Tyrode’s solution. The cells were then washed three times with Tyrode’s solution before fluorescence imaging using a halogen light with a 515 ± 35 nm bandpass excitation filter and a 590 nm high-pass emission filter. High-resolution optical mapping of action potentials (AP) was performed under a fluorescence microscope (Axio Vert. A1, Carl Zeiss) equipped with a high-speed CMOS camera (Zyla 4.2 PLUS, Andor, Belfast, UK). Data were collected at a sampling rate of 200 Hz and analysed using ImageJ (National Institutes of Health, 1.8.0_77).

### Patch-clamp recordings

Patch clamp was performed as previously described [17]. The action potentials were recorded using the whole-cell patch-clamp technique with the Axopatch 200B amplifier (Axon Instruments, Union City, CA, USA) at 37 °C. The prepared patch pipettes had a typical resistance of 3–6 MΩ using a micropipette puller (P-1000, Sutter Instrument, Novato, CA, USA). Action potential was recorded with an internal pipette solution, which contained the following (in mM): 120 K-aspartate, 25 KCl, 1.8 CaCl_2_, 5 Mg_2_ATP, 5 HEPES, 10 EGTA, and 10 glucose adjusted to pH 7.3 with KOH. The external bath solution contained the following (in mM): 135 NaCl, 5.4 KCl, 1.8 CaCl_2_, 1 MgCl_2_, 0.33 NaH_2_PO_4_, 10 HEPES, and 10 glucose adjusted to pH 7.3 with NaOH. Action potential amplitude (APA), average action potential duration (APD) at 90%, 50% repolarization of the amplitude (APD90, APD50) and resting membrane potential (RMP) were determined.

Calcium current (I_Ca_) was measured during the depolarization from a holding potential of −80 mV to test potentials ranging from −70 to +60 mV in 10 mV increments for 500 ms. I_Ca_ was recorded with the following internal solution designed to buffer the intracellular Ca^2+^ and eliminate the outward potassium current (I_K_) (in mM): 130 CsCl, 20 TEA-Cl, 1.8 MgCl_2_, 5 Na_2_ATP, 5 EGTA, 10 HEPES, and 10 glucose, adjusted to pH 7.3 with CsOH. The external bath solution contained the following (in mM): 120 choline chloride, 20 CsCl, 1.8 CaCl_2_, 1 MgCl_2_, 10 HEPES, and 10 glucose, adjusted to pH 7.3 with CsOH. I_Ca, L_ was recorded using the same solutions but from a holding potential of −40 mV.

I_K_ currents were recorded using an extracellular solution that contained the following (in mM): 135 NaCl, 5.4 KCl, 1 MgCl_2_, 0.33 NaH_2_PO_4_, 10 HEPES, and 10 glucose, adjusted to pH 7.2 with NaOH. The micropipettes were filled with an internal solution that contained the following (in mM): 45 KCl, 85 K-aspartate, 5 Na-pyruvate, 5 Mg_2_ATP, 10 EGTA, 10 HEPES, and 11 glucose, adjusted to pH 7.3 with KOH. To record the total K^+^ currents, the holding potential was set at −80 mV, and the cardiomyocytes were pre-stimulated at −110 mV for 150 ms followed by −50 mV for 50 ms to record the total I_K_. K^+^ currents were recorded at potentials ranging from −50 mV to +70 mV in 10 mV increments for 400 ms. All currents were normalized to the cell capacitance and expressed as pA/pF of the current density. Data were analysed with Clampfit 8.0 (Axon Instruments) and graphically described using SigmaPlot 10.0 (Systat Software, Inc., San Jose, CA, USA).

### Quantitative RT-PCR

RNA was prepared using the RNAprep pure Cell/Bacteria Kit (DP430, Tiangen, Beijing, China). RNA was reverse-transcribed into cDNA using iScript cDNA Synthesis Kit (170-8891, Bio-Rad, Hercules, CA, USA). Quantitative PCR was performed on the Applied Biosystems 7900HT Fast Real-Time PCR System (ABI) using SYBR Green (170-8882AP, Bio-Rad). The expression levels were normalized to the housekeeping gene glyceraldehyde 3-phosphate dehydrogenase (GAPDH). The oligonucleotide sequences are summarized in Additional file 1: Table S1.

### Western blot

Protein was extracted from the lysed VCMs as previously described [18]. The total protein concentration was determined using a Pierce™ BCA Protein Assay Kit (Thermo Fisher Scientific, Waltham, MA, USA, 23225). Equal amounts of protein were separated by electrophoresis on SDS-PAGE gels (Beyotime, P0012A) and then blotted onto nitrocellulose membranes. Then, the membranes were incubated with primary antibodies overnight. The following primary antibodies were used for the western blotting: GRP78 (1:1000, Cell Signaling, Danvers, MA, USA, 3177), CHOP (1:1000, Cell Signaling, 2895), calpain (1:1000, Cell Signaling, 2539), cTnT (1:1000, Cell Signaling, 5593), caspase-3 (1:1000, Cell Signaling, 9662), Bcl-2 (1:1000, Cell Signaling, 3498), Bax (1:1000, Cell Signaling, 2772), L-type calcium channel (1:1000, Abcam, ab96713), β-tubulin (1:1000, Cell Signaling, 2148) and GAPDH (1:1000, Cell Signaling, 2118S). Secondary antibodies used were anti-rabbit IgG, HRP-linked antibody (1:5000, Cell Signaling, 7074P2) and anti-mouse IgG, HRP-linked antibody (1:5000, Cell Signaling, 7076). After the addition of the chemiluminescent reagents (Thermo Fisher Scientific, 17295), the bands were detected using a Molecular Imager ChemiDoc™ XRS+ Imaging System (Bio-Rad) and quantified using the Image Lab™ Software (Bio-Rad).

### Statistical analysis

Unless stated otherwise, all data were expressed as the means ± SD. The statistical analysis was performed using SPSS Statistics 19.0 (IBM Corp., Armonk, NY, USA). Differences between the experimental groups were analysed by one-way ANOVA, followed by the LSD and SNK test. A *p* value of < 0.05 was considered significant for all statistical tests.

## Results

### Generation of in vitro ventricular pacing model

Human iPSC line (NC5) was cultured and differentiated to VCMs with small molecules treatment according to a previously described protocol [10]. These VCMs were maintained for approximately 6 weeks and then were treated with 1.2 Hz frequency, 0.5 ms duration, 3 V voltage electrical stimulations according to the initial parameter settings of pacemaker implantation [19]. The contactless optical mapping [16] confirmed that the average beating rate increased from approximately 36 bpm to approximately 71 bpm during the stimulation, indicating that the pacing capture efficiency was excellent (Additional file 2: Figure S1).

**Fig. 1:**
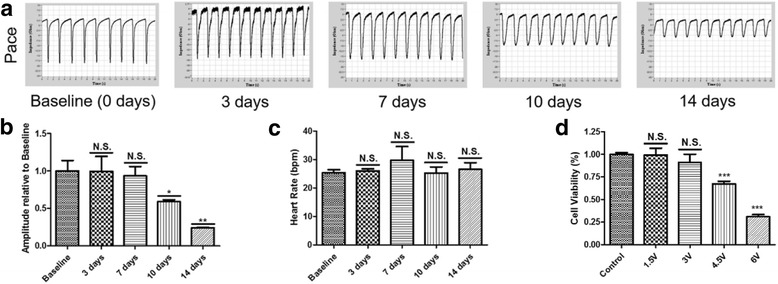
Long-term pacing led to a dramatic decrease in the beating spike amplitude. **a** Plots are representative of the spontaneous beating characteristics of the paced VCMs; **b** quantification of the beating spike amplitude demonstrated a dramatic decrease in the 10-day and 14-day paced VCMs compared with the baseline controls; **c** however, the spontaneous beating rate revealed no significant difference during the whole pacing process; **d** VCMs were exposed to 0.5 ms duration and 1.2 Hz frequency pulses with 0, 1.5, 3, 4.5, 6 V voltage for 2 weeks. Cell viability was measured with CCK-8 assay and the results were presented as the means ± SD of three independent experiments. ^*^ *p* < 0.05, ^***^ *p* < 0.001 according to paired a Student’s *t* test (Baseline/Control vs. each point)

To further confirm the time-dependent effects of pacing on VCMs, the impedance beating recordings were investigated as previously described [20]. Six-week-old VCMs were used as baseline controls. After continuous pacing for 3, 7, 10 and 14 days, VCMs were dissociated onto gelatin-coated 96-well impedance plates at 50,000 viable cells per plate. Parallel cultivation without stimulation was also seeded onto the plates. Impedance measurements of the contractions were recorded from spontaneously beating monolayers 2 days post-seeding using a CardioExcyte 96 system. Figure 1a plots the representative spontaneous beating characteristics of paced VCMs. It demonstrated a dramatic decrease in the beating spike amplitude in the 10-day and 14-day paced VCMs compared with the baseline control (Fig. 1b). However, the spontaneous beating rate revealed no significant difference during the whole pacing process (Fig. 1c). However, the beating patterns in the non-paced cells revealed no significant changes at any time point (Additional file 3: Figure S2). The results illuminated that pacing VCMs in vitro over 10 days caused cellular damage to a certain extent.

Subsequently, we investigated the effects of different stimulation voltage on cell viability. In detail, VCMs were exposed to 0.5 ms duration and 1.2 Hz frequency pulses with 0, 1.5, 3, 4.5, 6 V voltage for 2 weeks. Cell viability was measured with CCK-8 assay as previously described [13]. As shown in Fig. 1d, 4.5 V and 6 V voltage stimulation gave rise to 32.7% and 69.1% reduction of cell viability (*p* < 0.001), indicating that strong pacing conditions may lead to direct physical injury on VCMs. Thus, 0.5 ms duration, 3 V voltage and 1.2 Hz frequency pulses were selected as proper parameters to study the deleterious effects of long-term pacing.

### Long-term pacing disrupted the contractile structures of cardiomyocytes

**Fig. 2:**
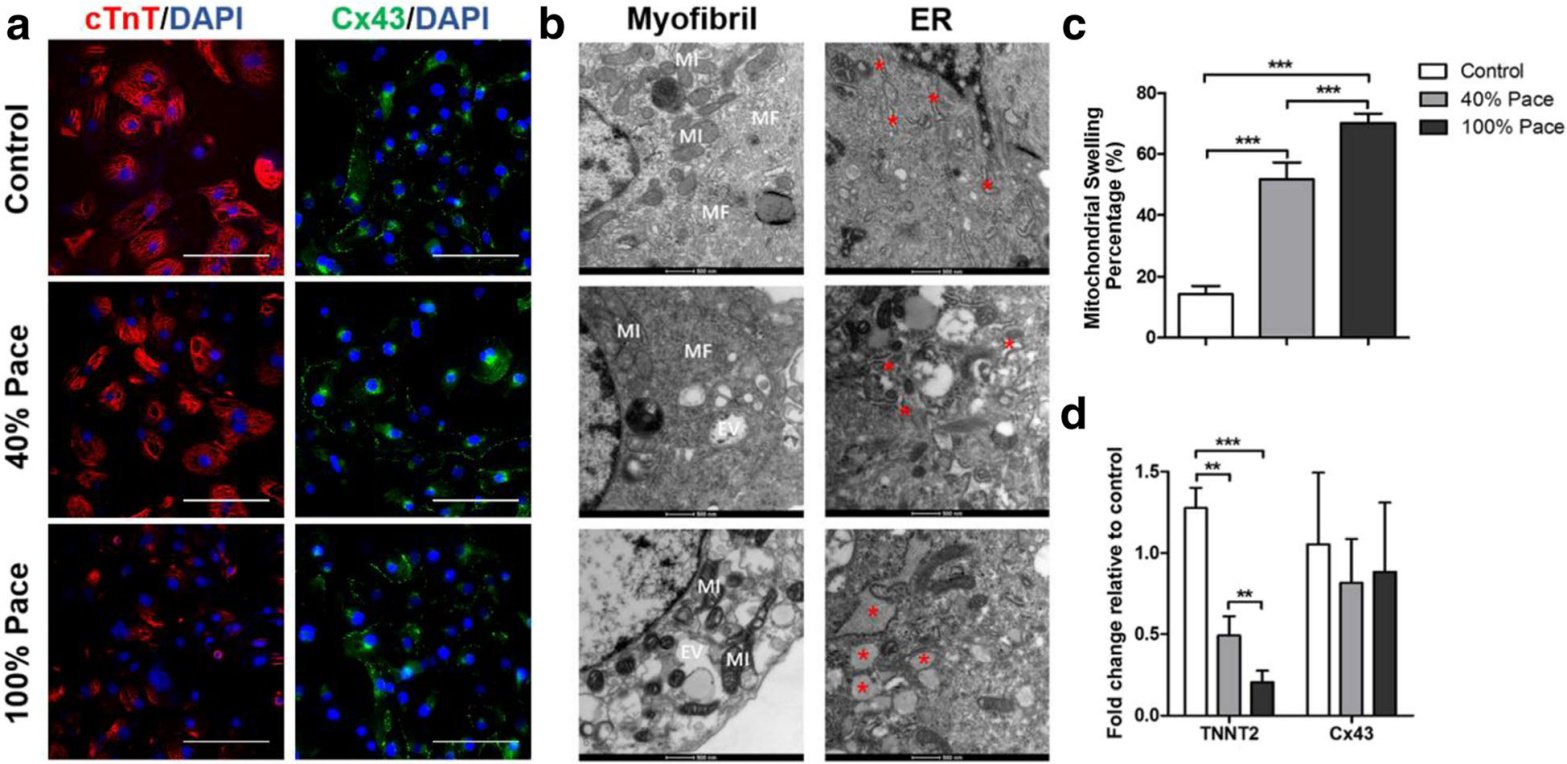
Long-term pacing disrupted the contractile structure of cardiomyocytes. **a** Compared with age-matched controls, the paced VCMs demonstrated decreased expression of cTnT, while cells from both the pacing and control groups showed robustly expressed Cx43 (scale bar 50 μm). TEM investigation illustrated significantly increased intracellular empty vacuoles and decreased myofibril density in paced VCMs (*EV* empty vacuoles, *MF* myofibrils, *MI* mitochondria, scale bar 500 nm, Myofibril panel, **b**); pacing also significantly increased the swelling mitochondria percentage, **c**; endoplasmic reticula (*ER*) with narrow cisterns were observed in the controls. By contrast, the paced VCMs demonstrated significantly increased aberrant dilated electron-dense ERs, which were swollen into circular shapes (^*^endoplasmic reticulum, scale bar 500 nm, ER panel, **b﻿**); qRT-PCR investigation revealed that the expression of TNNT2 was significantly lower in the pacing groups compared with the age-matched controls. However, the expression of Cx43 demonstrated a similar level among 40% Pace, 100% Pace and control VCMs (**d**). ^**^ *p* < 0.005, ^***^ *p* < 0.001 according to a one-way ANOVA test

A previous study has reported that long-term ventricular pacing has deleterious effects and becomes more significant when cumulative percent ventricular pacing (Cum%VP) exceeds 40% of time [4]. To investigate the effects of different Cum%VP on cardiomyocytes, we utilized two different protocols in which the cumulative percent pacing was set to 40% (approximately 10 h/day) or 100% (24 h/day) with a 1.2 Hz frequency, 0.5 ms duration, 3 V voltage electrical stimulation. Parallel cultivation without stimulation was also performed as a control. After 2 weeks of stimulation, the cells were digested and immunostained. The results showed that compared with age-matched controls (approximately 8 weeks), pacing substantially decreased the expression of cardiac troponin T (cTnT) characterized by a poor contractile machinery composed of misaligned myofibrils at a low density (Fig. 2a). However, cells from both pacing and control groups robustly expressed connexin 43 (Cx43).

The ultrastructural elements underlying the morphological changes were observed by TEM. Consistent with the findings in the confocal images, TEM illustrated that long-term pacing significantly increased the intracellular empty vacuoles and decreased the myofibrillar density, thereby disrupting the myofibril ultrastructural organization (Fig. 2b). Pacing also significantly increased the swelling mitochondria percentage (Control vs. 40% Pace vs. 100% Pace, 14.14 ± 2.83% vs. 51.62 ± 5.67% vs*.* 70.20 ± 3.13%, *p* < 0.001, Fig. 2c) compared with the age-matched controls. These results were consistent with the myofibrillar variation and mitochondrial morphological changes in endomyocardial biopsies from patients following chronic ventricular pacing [7]. Moreover, a few strands of rough endoplasmic reticula (ER) with narrow cisterns were observed in the controls. By contrast, the paced VCMs demonstrated significantly increased aberrant dilated electron-dense ERs, which were swollen into circular shapes, suggesting the potential activation of ER stress (Fig. 2b). Furthermore, gene expressions in the cardiac structures (TNNT2 and Cx43) were investigated by quantitative RT-PCR (Fig. 2d). The expression of TNNT2 was significantly lower in the 40% Pace and 100% Pace groups (1.28 ± 0.10 vs. 0.49 ± 0.10 vs. 0.20 ± 0.06 *fold change relative to the control*, *p* < 0.001). However, the expression level of Cx43 was similar among 40% Pace, 100% Pace and control cardiomyocytes. The results showed that long-term pacing disrupted the contractile structure of VCMs, which was dependent on the Cum%VP.

### Long-term pacing increased the cardiac apoptosis ratio

**Fig. 3:**
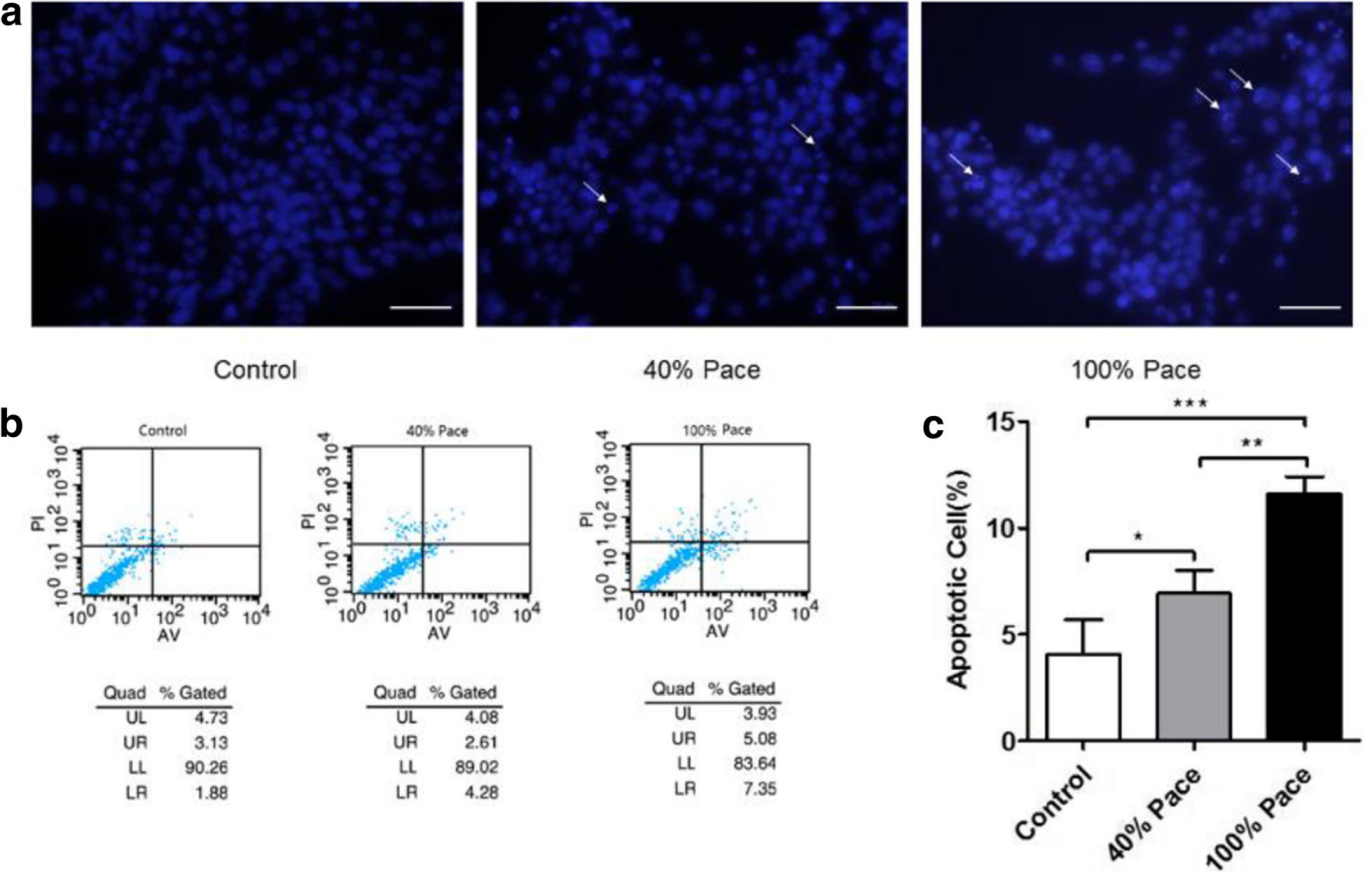
Long-term pacing induced the cardiac apoptosis. **a** Hoechst 33342 staining demonstrated that the intact nuclei containing aequalis chromatin were homogeneously distributed in the controls. By contrast, as the Cum%VP increased, the VCMs exhibited typical morphological features of apoptosis as revealed by shrunken cells with condensed or fragmented nuclei (*arrow*: apoptosis cell, scale bar 50 μm); cytometric analysis with annexin V and propidium iodide (*AV/PI*) double staining was further conducted to quantify the occurrence of apoptosis (**b**). The results illuminated that pacing significantly increased the apoptosis ratio in a Cum%VP-dependent manner (**c**). ^*^ *p* < 0.05, ^**^ *p* < 0.005, ^***^ *p* < 0.001 according to a one-way ANOVA test

Investigation of the morphological patterns revealed that long-term pacing dramatically induced mitochondrial and endoplasmic reticular morphological changes. Our previous study suggested that mitochondria and ERs play a central role in integrating apoptotic pathways [13]. We then assessed the morphological features of the cell nuclei by Hoechst 33342 staining. The results demonstrated that intact nuclei containing aequalis chromatin were homogeneously distributed in control VCMs. By contrast, as the Cum%VP increased, the VCMs exhibited typical morphological features of apoptosis, such as shrunken cells with condensed or fragmented nuclei (Fig. 3a). The results demonstrated that the cells were undergoing apoptosis. To quantify the occurrence of apoptosis, we then carried out a cytometric analysis with annexin V and propidium iodide (AV/PI) double staining. As shown by the FACS analysis, the fourth and first quadrant cells represented early apoptotic cells (AV+/PI-) and late apoptotic cells (AV-/PI+), respectively (Fig. 3b). The results demonstrated that pacing significantly increased the apoptosis ratio in a Cum%VP-dependent manner (Control vs. 40% Pace vs*.* 100% Pace, 4.07 ± 1.63% vs*.* 6.92 ± 1.09% vs*.* 11.62 ± 0.81%, Fig. 3c).

### Long-term pacing disturbed the electrophysiological properties

**Fig. 4:**
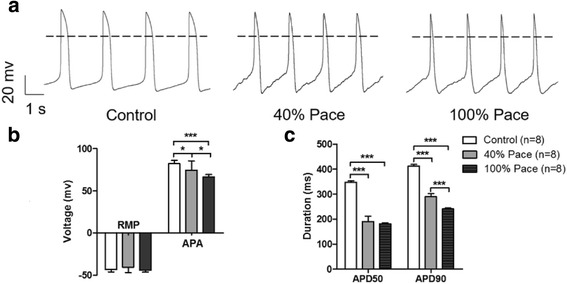
Long-term pacing remodelled the cardiac action potential. **a** Plots of representative APs in VCMs; quantification of the resting membrane potential and action potential amplitude (mean ± SD, n = 8, **b**) were performed. The paced iPSC-CMs demonstrated significantly shorter APD50 and APD90 (mean ± SD, n = 8, **c**) than the age-matched controls. *APA* action potential amplitude, *APD* average action potential duration, *RMP* resting membrane potential. ^*^ *p* < 0.05, ^**^ *p* < 0.005, ^***^ *p* < 0.001 according to a one-way ANOVA test

To evaluate the effects of pacing on the electrophysiological properties, the cardiomyocytes were dissociated for the electrophysiological analysis using the patch-clamp technique. Consistent with our previous findings [21], the action potentials (APs) revealed a homogeneous phenotype in which nearly 100% of the cells displayed typical ventricular-like AP parameters (Fig. 4a). The maximal depolarization amplitude exhibited a dramatic decrease from 82.24 ± 3.72 mV in control iPSC-CMs to 74.54 ± 10.18 mV (*p* < 0.05) in 40% Pace VCMs and 66.38 ± 2.77 mV (*p* < 0.001) in 100% Pace VCMs (Fig. 4b). Chronic electrical stimulation has been reported to shorten cardiac APs [22]. Consistent with previous findings, the shortening of phase 2 of the AP in the pacing VCMs was more obvious. Hence, AP durations measured at 50% and 90% repolarization from the AP peak (APD50 and APD90, respectively, Fig. 4c) were further analysed. The paced VCMs exhibited significantly shorter APD50 (Control vs. 40% Pace vs*.* 100% Pace, 348.27 ± 15.44 ms vs. 190.81 ± 59.36 ms vs. 181.38 ± 12.42 ms) and APD90 (Control *vs.* 40% Pace vs. 100% Pace, 412.18 ± 21.81 ms vs. 290.38 ± 33.45 ms *vs.* 241.10 ± 9.06 ms) than the age-matched controls.

**Fig. 5:**
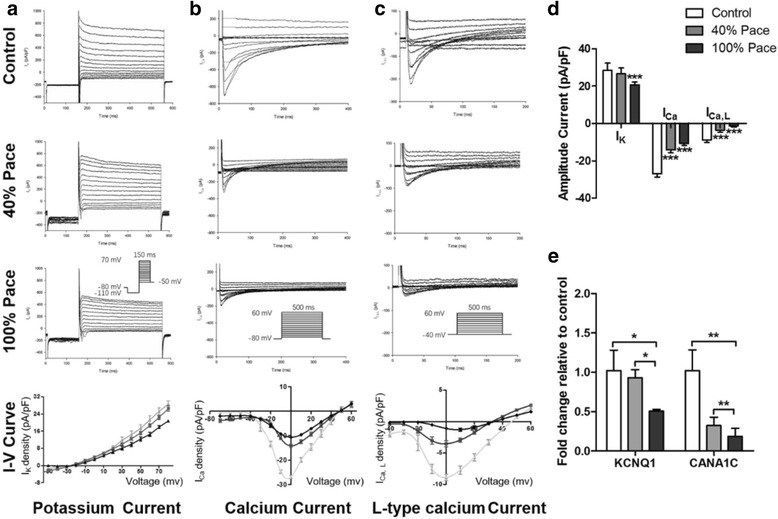
Long-term pacing disturbed ion currents in cardiomyocytes. Patch-clamp studies revealed that the K^+^ currents (**a**) were significantly lower in the 100% Pace group than those in controls (mean ± SD, n = 6, **d**); both calcium current (*I*_*Ca*_) (**b**) and L-type calcium current (*I*_*Ca, L*_) (**c**) were significantly lower in paced VCMs than those in controls (mean ± SD, n = 6, **d**); qRT-PCR demonstrated that the expression of KCNQ1 and CACNA1C was significantly lower in the 40% Pace and 100% Pace groups (**e**). ^*^ *p* < 0.05, ^**^ *p* < 0.005, ^***^ *p* < 0.001 according to a one-way ANOVA test

To further explain the shortening of phase 2 of the AP, we used voltage clamp recordings to measure the I_K_, I_Ca_ and L-type calcium (I_Ca, L_) currents. It was demonstrated that the potassium currents were significantly lower in the 100% Pace group compared with those in controls (I_K_ peak current density, Control vs. 40% Pace vs. 100% Pace, 28.54 ± 3.75 pA/pF vs. 26.59 ± 3.20 pA/pF vs. 20.76 ± 1.40 pA/pF, *p* < 0.001, n = 6, Fig. 5a, d). Furthermore, I_Ca_ amplitude in the pacing group dramatically reduced compared with controls (I_Ca_ peak current density, Control vs*.* 40% Pace vs. 100% Pace, -26.91 ± 1.51 pA/pF vs*.* -14.14 ± 1.37 pA/pF vs*.* -10.59 ± 1.09 pA/pF, *p* < 0.001, n = 6, Fig. 5b, d). Consistently, VCMs in the pacing groups had a lower I_Ca, L_ amplitude than controls (I_Ca, L_ peak current density, Control vs. 40% Pace vs. 100% Pace, -8.89 ± 1.30 pA/pF vs*.* -3.53 ± 1.13 pA/pF vs. -1.28 ± 0.61 pA/pF, *p* < 0.001, n = 6, Fig. 5c, d). Previous work has suggested that I_Ca_ plays an important role in the plateau of cardiac action potentials [23]. The differences depicted in ion channel studies are consistent with the APD changes. Furthermore, the expression of the major cardiac ion-channel genes KCNQ1 and CACNA1C were studied using quantitative RT-PCR. The results showed that the expression levels of KCNQ1 and CACNA1C were significantly lower in the 40% and 100% Pace groups (KCNQ1, 1.02 ± 0.26 vs*.* 0.93 ± 0.10 *vs.* 0.51 ± 0.02 *fold change relative to the control*, *p* < 0.05; CACNA1C, 1.02 ± 0.26 vs. 0.32 ± 0.11 vs. 0.19 ± 0.10 *fold change relative to the control*, *p* < 0.005, Fig. 5e). These results were consistent with the alterations in the potassium and calcium channels observed in cardiac biopsies of long-term pacing patients [8].

### Long-term pacing induced ER stress and calpain activity

**Fig. 6:**
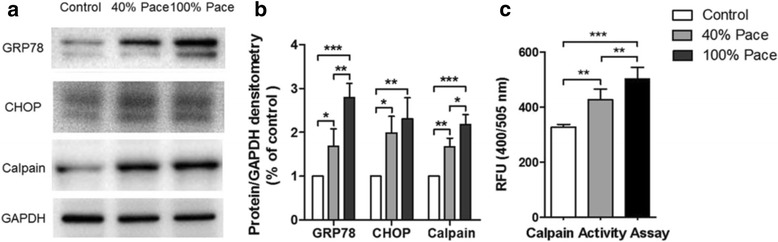
Long-term pacing induced ER stress and calpain activity. Western blot (**a**) analysis demonstrated that the protein levels of both GRP78 and CHOP were markedly elevated after pacing compared to those in the age-matched controls (**b**), indicating the occurrence and exacerbation of ER stress. Consistently, it also demonstrated a significantly higher expression level of calpain in the paced VCMs than in the controls. The calpain activity was further investigated by an ELISA assay. The results revealed that pacing induced calpain activity in a Cum%VP-dependent manner (**c**). ^*^ *p* < 0.05, ^**^ *p* < 0.005, ^***^ *p* < 0.001 according to a one-way ANOVA test

Organelle morphology analysis using TEM suggested a potential activation of ER stress. We therefore examined the protein expression of several ER stress markers by western blotting. The release of ER chaperon GRP78 and enhanced expression of CHOP are generally associated with the occurrence and exacerbation of ER stress [24]. Western blot analysis demonstrated that the protein levels of both GRP78 and CHOP were markedly elevated after pacing compared to those in the age-matched controls (Fig. 6a, b). As previous studies have reported that the overactivation of calpain, which is a Ca^2+^-dependent neutral protease, by ER stress could induce cell death in ischaemic brain injury [25], liver disease [26], and atrial fibrillation [27], the protein expression of calpain was then assessed in our study. The results demonstrated significantly higher levels of calpain in 40% and 100% Pace groups compared with those in the controls. In addition, the relative fluorescence units (RFU) in the Calpain Activity Assay (400/505 nm) were 327.33 ± 8.38 in the control group but increased to 427.17 ± 35.46 in 40% Pace group and 502.67 ± 38.02 in 100% Pace group (Fig. 6c), suggesting that calpain was activated in a Cum%VP-dependent manner.

### Inhibition of calpain activity attenuated the adverse effects of pacing

Previous studies have shown that activated calpain mediated the degradation of myofibrillar structure [27], cardiomyocyte loss [28] and I_Ca, L_ reduction [29] in the pressure-overloaded feline myocardium and atrial fibrillation models. Given our results showing structural and electrical remodelling in the paced VCMs, we hypothesized that the inhibition of calpain may attenuate the adverse effects of pacing.

**Fig. 7:**
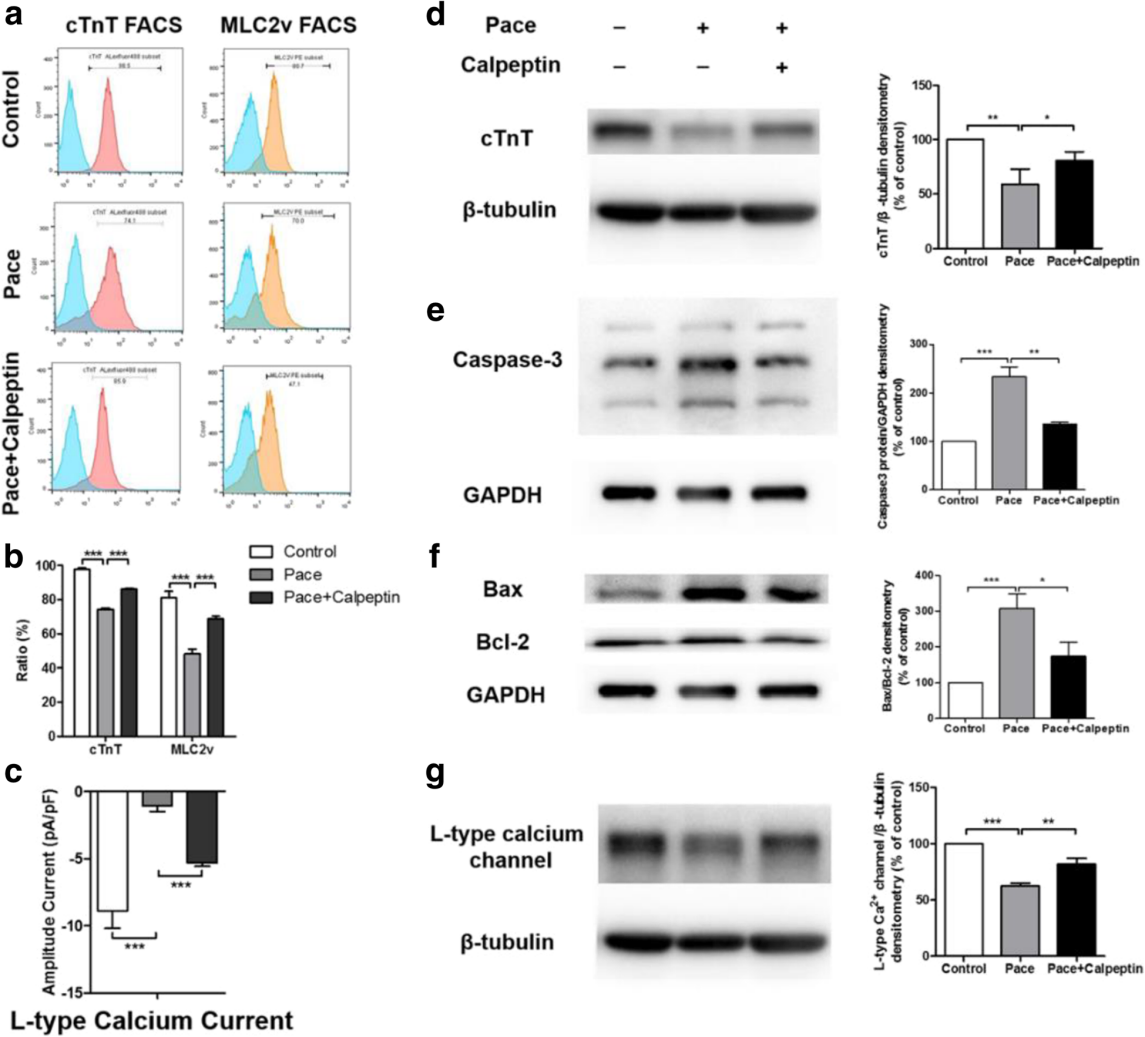
Inhibition of calpain activity attenuated the adverse effects of pacing. Flow cytometry analysis of cardiac troponin T (cTnT) and MLC2v (**a**) demonstrated that the pharmaceutical inhibition of calpain activation significantly increased the cTnT^+^ and MLC2v^+^ cells ratio compared with that in the paced VCMs (**b**). Patch-clamp studies revealed that I_Ca, L_ density was significantly increased after the calpeptin treatment (**c**). In the calpeptin (5 μM)-treated group, the protein levels of cTnT (**d**) and L-type calcium channel (**g**) were increased by western blot analysis. The expression of apoptosis proteins (caspase-3, **e**; Bax/Bcl-2, **f**) that are involved in ER stress decreased markedly upon the calpeptin treatment compared with 100% Pace group. ^*^ *p* < 0.05, ^**^ *p* < 0.005, ^***^ *p* < 0.001 according to a one-way ANOVA test

We thus examined the effects of the calpain inhibitor calpeptin on the 100% paced cells. We found that treatment with calpeptin (5 μM) restored the cTnT^+^ cell ratio in the 100% Pace group to a level that was similar to that observed in the control VCMs (Control vs*.* Pacing vs. Pacing + Calpeptin, 97.60 ± 0.85% vs. 74.20 ± 0.75% vs. 86.13 ± 0.40%, Fig. 7a, b). Previous studies have suggested that there is a direct and early role of MLC2v phosphorylation in regulating actin-myosin interactions in striated muscle contraction, and loss of these mechanisms could play a critical role in heart failure [30]. Further FACS analyses of MLC2v demonstrated that calpeptin (5 μM) preserved the MLC2v^+^ cells ratio compared to that in the 100% paced cells (Fig. 7a, b), indicating diminishing degradation of myofibril structure. Consistent with the FACS analysis, western blot analysis demonstrated that the protein level of cTnT was markedly decreased after pacing compared to that in the age-matched controls, but the addition of calpeptin significantly alleviated this change (Fig. 7d), indicating that the inhibition of calpain suspended the structural remodelling in the paced VCMs. Moreover, the results of the western blot analysis showed that the expression of apoptosis proteins (caspase-3, Bax/Bcl-2) that are involved in ER stress decreased markedly in the calpeptin (5 μM)-treated group compared with that in the 100% Pace group (Fig. 7e, f).

Furthermore, we assessed the effects of inhibition of calpain activity on the electrophysiological properties. After the calpeptin treatment, there was a significant increase in the I_Ca, L_ density in the paced VCMs as detected by patch clamp (Fig. 7c, Additional file 4: Figure S3a, b). Furthermore, the calpeptin-treated VCMs exhibited a significantly increased level of L-type calcium channel expression compared with 100% paced VCMs (Fig. 7g). Taken together, these findings illustrated that the inhibition of calpain could attenuate the adverse effects of pacing.

## Discussion

Based on various clinical trials, it has become apparent that a high amount of RV apical pacing may be associated with a poor clinical outcome [31]. Even dual-chamber pacing (DDD/R), which is adopted as the “physiological” pacing mode by maintaining AV synchrony, does not reduce death compared with single-chamber ventricular pacing (VVI/R) after many years of follow-up [4]. Using magnetic resonance imaging, Prinzen et al. noted myocardial strain and metabolic changes in an animal model of cardiac pacing [32]. Spragg et al. provided evidence that an altered activation sequence may induce changes in protein expression levels that may have adverse effects on ventricular function in a canine pacing model [33]. Although animal studies have yielded significant insights into VP-induced ventricular remodelling at the molecular level, they also suffer from several limitations that prevent a precise modelling of human cardiac disease. For example, the electrophysiological properties, ion channel contributions, and cardiac development of the animal myocardium are all different from those of humans [34]. In addition, primary rat cardiomyocytes and cardiac tissues from paced patients do not survive in long-term cultivation, adding to the difficulty in building a longer-lasting (lasting longer than weeks) pacing model in vitro. Consequently, thus far, only limited evidence has been discovered regarding the functional disturbances caused by VP and the underlying mechanisms.

Generation of iPSC-CMs provides abundant sources for disease modelling [35], drug screening [16] and cell replacement therapy [36], thereby circumventing the limitations that are associated with animal models. In the present study, human iPSC-derived VCMs were cultured in the presence of electrical stimulation for 2 weeks. Quantitative structural and electrophysiological analyses were used to define the functional disturbances of VP. Compared to controls, paced VCMs exhibited a remarkable reduction in contractile protein expression, an increased apoptosis ratio and electrophysiological remodelling. Notably, the morphological changes strongly indicated the activation of ER stress. In fact, a universal upregulation in the proteins that are involved in ER stress was confirmed by western blotting and an ELISA assay, further demonstrating the activation of downstream calpain. Moreover, treatment with the calpain inhibitor (calpeptin) could attenuate the adverse effects of the structural remodelling and increased I_Ca, L_ in the paced VCMs, implicating ER stress and downstream calpain activity as possible regulatory mechanisms underlying the pathogenesis of the disease.

It is widely acknowledged that newly differentiated cardiomyocytes are in a foetal state with immature structural and electrophysiological properties [37]. Previously, electrical stimulation has been introduced to promote cardiogenesis [38] or maturation [39] in cultured embryoid bodies derived from human embryonic stem cells. However, these positive effects are reported in early-stage cardiomyocytes (within approximately 2 weeks) with short stimulation times (<10 days) [40]. On the other hand, ultrastructural [41] and electrophysiological [42] maturation of cardiomyocytes has been described using prolonged cultivation (where the cardiomyocytes were maintained in in vitro culture for more than 30 days). In the present study, we stimulated 6-week-old, relatively mature VCMs with 1.2 Hz frequency, 0.5 ms duration, 3 V voltage electric pulses. Consistent with previous findings [39], the beating spike amplitude revealed no significant changes in the first 7 days by CardioExcyte 96. However, the results revealed a dramatic decrease in the amplitude in the 10-day and 14-day paced VCMs compared with the age-matched controls, indicating adverse effects by long-term pacing. We further electrically stimulated the VCMs in vitro for 2 weeks to generate a long-term pacing model. A previous study has described dystrophic calcifications, disorganized mitochondria and myofibrillar cellular disarray in a canine pacing model [43]. Consistently, the paced VCMs displayed decreased myofibrillar alignments, edematous mitochondria and aberrant dilated ERs compared with the age-matched controls. We further demonstrated that long-term pacing increased the cardiac apoptosis ratio using Hoechst 33342 staining and flow cytometric analysis. Moreover, the patch-clamp recordings of the paced VCMs demonstrated a reduced APA, shortened APD, and reduced I_K_, I_Ca_ and I_Ca, L_ compared to those in the age-matched controls, which were all consistent with the patch-clamp findings in the canine pacing model [44, 45]. The results illustrated that pacing relatively mature VCMs in vitro for 2 weeks led to a series of structural and electrophysiological dysfunctions, which was a great tool for identifying new therapeutic targets for the management of long-term pacing patients.

The investigation of the protein expressions illustrated that long-term pacing universally activated ER stress and downstream calpain. Considering that activated calpain mediated the degradation of myofibrillar structure [27], cardiomyocyte loss [28] and I_Ca, L_ reduction [29], we hypothesized that the inhibition of calpain may attenuate the adverse effects of pacing. Cardiac troponin T was reported to be a marker of cardiomyocytes [46]. In addition, MLC2v was proven to be an adequate ventricular marker in humans [47]. Recently, Burridge and colleagues have shown that MLC2v positivity consistently increased during the time course, while unspecified cardiomyocyte precursors demonstrated an MLC2v^−^ phenotype at the beginning of differentiation, suggesting that the presence of MLC2v^+^ cells was boosted in parallel with the myofibril development [48]. In the present study, compared with the paced VCMs, the pharmaceutical inhibition of calpain activation significantly increased the cTnT^+^ and MLC2v^+^ cells ratios, suggesting that the suspension of the pacing induced structural remodelling. Western blot analysis further confirmed that the addition of calpeptin significantly alleviated the expression changes in cTnT and apoptosis proteins induced by long-term pacing. Consistently, we demonstrated that treatment with calpeptin significantly increased the I_Ca, L_ density and channel expression in the paced VCMs. Taken together, our results identified a potential therapeutic target for disease management. Moreover, it was shown that long-term pacing presents deleterious effects in a Cum%VP-dependent manner, suggesting that an optimizing pacemaker programme to reduce ventricular events could be beneficial for controlling the adverse effects of long-term pacing.

## Conclusions

In summary, our data indicated that long-term electrical stimulation on VCMs caused impairments in myofilament regulation, increased apoptosis ratio, and electrophysiological remodelling in a Cum%VP-dependent manner, which might be the primary reason for the eventual appearance of the pacing-induced adverse events in patients. Our findings demonstrated that ER stress and downstream calpain could be central mechanisms that underlie disease pathogenesis. The pharmacological inhibition of calpain activity suspended the development of structural and electrophysiological irregularities, suggesting that this inhibition might be a novel therapeutic target for disease management.

## Additional files

Additional file 1: Table S1. List of oligonucleotide sequences used. (DOC 237 kb)

Additional file 2: Figure S1. The contactless optical mapping displayed action potential prolongation *in situ*. It was confirmed that the average beating rate increased from 36.1 bpm to 71.0 bpm during stimulation, indicative of the excellent pacing capture efficiency. (DOC 102 kb)

Additional file 3: Figure S2. Beating patterns in non-paced cells revealed no significant changes at different time points. (a) Plots representative of the spontaneous beating characteristics of VCMs; the spontaneous beating spike amplitude (b) and beating rate (c) revealed no significant difference during the whole process. (DOC 206 kb)

Additional file 4: Figure S3. Patch-clamp investigation of I_Ca, L_ after calpeptin treatment (a); further statistical analysis revealed that drug treatment attenuated the adverse effects on I_Ca, L_ in paced VCMs (b). (DOC 118 kb)

## Abbreviations

AP: Action potential
APA: Action potential amplitude
APD: Action potential duration
Cum%VP: Cumulative percent ventricular pacing
ER: Endoplasmic reticulum
I_Ca_: Calcium current
I_Ca, L_: L-type calcium current
ICD: Implantable cardioverter-defibrillator
I_K_: Potassium current
iPSC: Induced pluripotent stem cell
RV: Right ventricular
TEM: Transmission electron microscopy
VCM: Ventricular cardiomyocyte
