# Supplementary figures and images for: Structural and electrophysiological dysfunctions due to increased endoplasmic reticulum stress in a long-term pacing model using human induced pluripotent stem cell-derived ventricular cardiomyocytes
Source: Stem Cell Res Ther. 2017 May 11;8:109. doi: 10.1186/s13287-017-0566-6 (PMC5426064; doi:10.1186/s13287-017-0566-6)

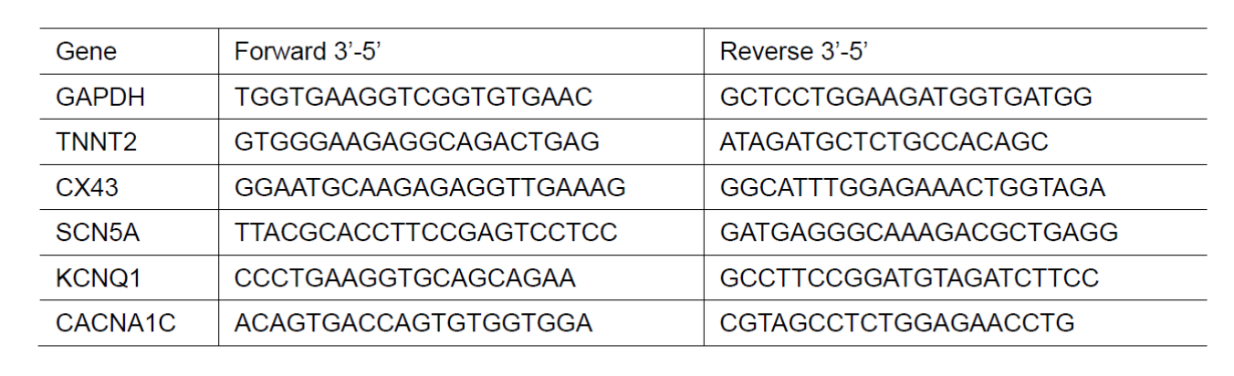


**Table S1.** List of Oligonucleotide sequences used.

Supplement: Supplementary file 1 — List of oligonucleotide sequences used. (DOC 237 kb) [file 13287_2017_566_MOESM1_ESM.doc]
