# Supplementary material for: Structural and electrophysiological dysfunctions due to increased endoplasmic reticulum stress in a long-term pacing model using human induced pluripotent stem cell-derived ventricular cardiomyocytes
Source: Stem Cell Res Ther. 2017 May 11;8:109. doi: 10.1186/s13287-017-0566-6 (PMC5426064; doi:10.1186/s13287-017-0566-6)
Supplement: Supplementary file 2 — The contactless optical mapping displayed action potential prolongation in situ. It was confirmed that the average beating rate increased from 36.1 bpm to 71.0 bpm during stimulation, indicative of the excellent pacing capture efficiency. (DOC 102 kb) [file 13287_2017_566_MOESM2_ESM.doc]

**
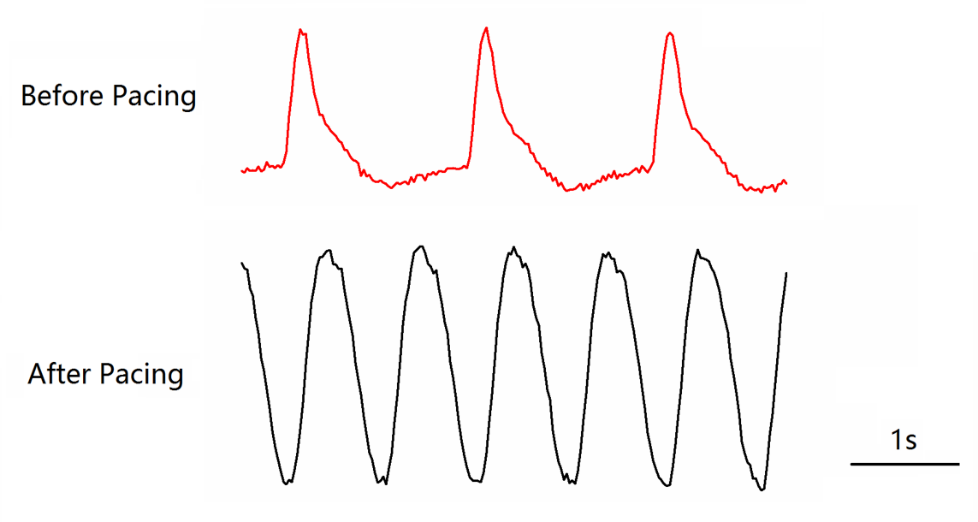
**

**Figure S1.** The contactless optical mapping displayed action potential prolongation in situ. It was confirmed that the average beating rate increased from 36.1 bpm to 71.0 bpm during stimulating, indicative of the excellent pacing capture efficiency.
