# Supplementary material for: Structural and electrophysiological dysfunctions due to increased endoplasmic reticulum stress in a long-term pacing model using human induced pluripotent stem cell-derived ventricular cardiomyocytes
Source: Stem Cell Res Ther. 2017 May 11;8:109. doi: 10.1186/s13287-017-0566-6 (PMC5426064; doi:10.1186/s13287-017-0566-6)
Supplement: Supplementary file 3 — Beating patterns in non-paced cells revealed no significant changes at different time points. (a) Plots representative of the spontaneous beating characteristics of VCMs; the spontaneous beating spike amplitude (b) and beating rate (c) revealed no significant difference during the whole process. (DOC 206 kb) [file 13287_2017_566_MOESM3_ESM.doc]

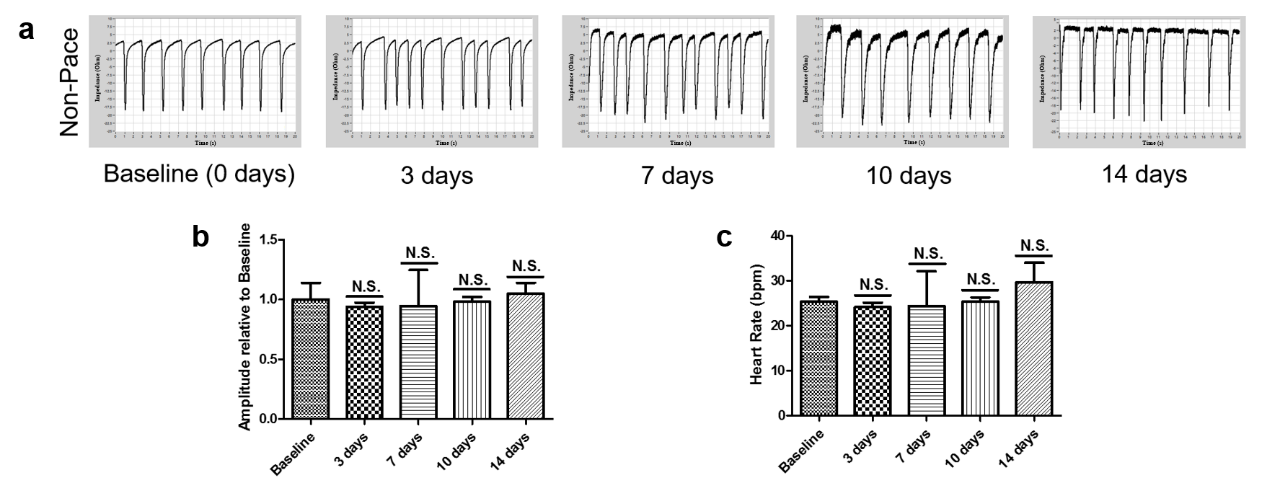


**Figure S2.** Beating patterns in non-paced cells revealed no significant changes at different time points. (a) plots representative spontaneous beating characteristics of VCMs; the spontaneous beating spike amplitude (b) and beating rate (c) revealed no significant difference during the whole process.
