# Supplementary material for: Structural and electrophysiological dysfunctions due to increased endoplasmic reticulum stress in a long-term pacing model using human induced pluripotent stem cell-derived ventricular cardiomyocytes
Source: Stem Cell Res Ther. 2017 May 11;8:109. doi: 10.1186/s13287-017-0566-6 (PMC5426064; doi:10.1186/s13287-017-0566-6)
Supplement: Supplementary file 4 — Patch-clamp investigation of ICa, L after calpeptin treatment (a); further statistical analysis revealed that drug treatment attenuated the adverse effects on ICa, L in paced VCMs (b). (DOC 118 kb) [file 13287_2017_566_MOESM4_ESM.doc]

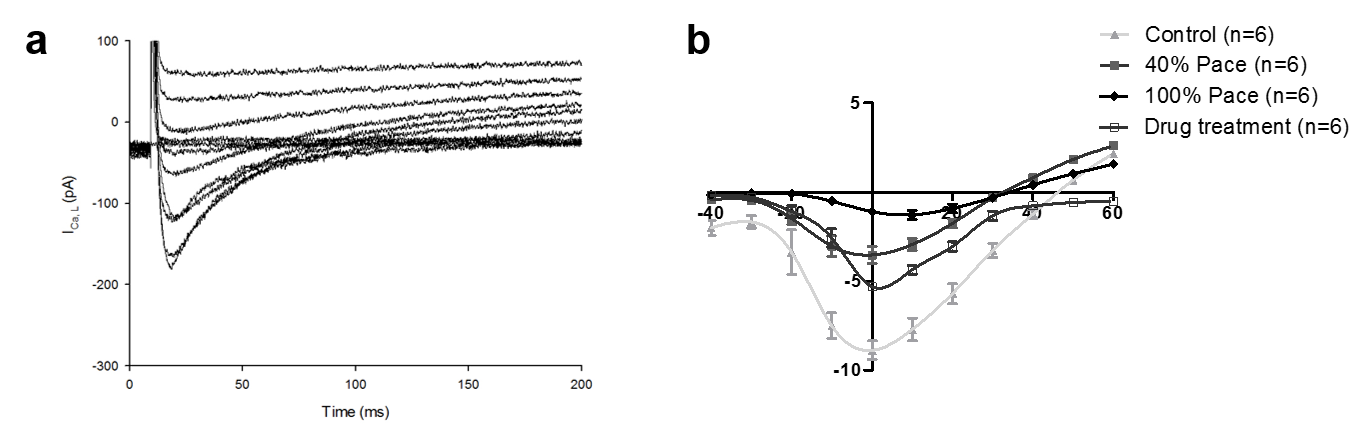


**Figure S3.** Patch clamp investigation of I_Ca, L_ after calpeptin treatment (a); further statistical analysis revealed that drug treatment attenuated the adverse effects on I_Ca, L_ in paced VCMs (b).
